# Pecbloodin_18-37_: a promising antimicrobial peptide from *Boleophthalmus pectinirostris* with therapeutic potential against *Edwardsiella tarda* infection

**DOI:** 10.1128/aem.02043-25

**Published:** 2026-02-23

**Authors:** Yuqi Bai, Wenbin Zheng, Weibin Zhang, Jingyuan Zhan, Fangyi Chen, Ke-Jian Wang

**Affiliations:** 1 State Key Laboratory of Marine Environmental Science, College of Ocean & Earth Sciences, Xiamen University 12466, Xiamen, Fujian, China; 2 State-Province Joint Engineering Laboratory of Marine Bioproducts and Technology, College of Ocean & Earth Sciences, Xiamen University 12466, Xiamen, Fujian, China; 3 Innovation Research Institute for Marine Biological Antimicrobial Peptide Industry Technology, Fujian Ocean Innovation Center 12466, Xiamen, China; Washington University in St. Louis, St. Louis, Missouri, USA

**Keywords:** *Edwardsiella tarda*, mudskipper, innate immunity, Pecbloodin_18-37_, antimicrobial peptide

## Abstract

*Edwardsiella tarda* is a significant pathogen in aquaculture, causing severe systemic infections and considerable economic losses worldwide. The limited efficacy of current treatments and the emergence of multidrug-resistant strains underscore the urgent need for novel antimicrobial strategies. Antimicrobial peptides have garnered increasing interest due to their broad-spectrum activity and low risk of resistance development. In this study, we identified a novel immune-related gene from the mudskipper *Boleophthalmus pectinirostris*, named *Pecbloodin*, which encodes a mature peptide of 67 amino acids. A truncated peptide derived from this sequence, Pecbloodin_18-37_, exhibited broad-spectrum antibacterial activity, high thermal stability, and tolerance to sodium ions. Functional assays demonstrated that Pecbloodin_18-37_ exerts rapid bactericidal effects by compromising bacterial membrane integrity, inducing cellular content leakage, and triggering endogenous reactive oxygen species accumulation. Furthermore, it effectively inhibited bacterial biofilm formation and did not promote resistance under prolonged exposure. Importantly, *in vivo* experiments using an *E. tarda*-infected mudskipper model revealed that Pecbloodin_18-37_ significantly improved host survival and modulated the immune response. Overall, Pecbloodin_18-37_ shows great potential as a promising alternative to conventional antibiotics for the control of *E. tarda* infections in aquaculture, addressing the pressing issue of antibiotic resistance.

**IMPORTANCE:**

*Edwardsiella tarda* is an urgent threat to global aquaculture. We mined the mudskipper *Boleophthalmus pectinirostris* genome for antimicrobial peptide and identified Pecbloodin_18-37_, a 20-aa thermostable peptide that rapidly permeabilizes bacterial membranes, elicits intracellular reactive oxygen species, blocks biofilm formation, and does not select for resistance. In *E. tarda*-challenged fish, a single dose reduced mortality by 25% and restored immune homeostasis. The peptide is readily synthesized and feed-compatible, providing an immediate, resistance-proof substitute for conventional antibiotics in fish farming.

## INTRODUCTION

Bacterial pathogens, such as *Edwardsiella*, *Aeromonas*, *Vibrio*, and *Pseudomonas* species, are widespread in aquaculture systems and can rapidly cause mass mortality events, leading to substantial economic losses estimated at approximately 6 billion USD annually (1). Among them, the genus *Edwardsiella* encompasses several important pathogens, including *Edwardsiella tarda*, *Edwardsiella ictaluri*, *Edwardsiella piscicida*, and *Edwardsiella anguillarum*, which pose significant threats to both aquaculture and public health on a global scale (2). Notably, *E. tarda* is the only species within this genus known to infect humans. It is ubiquitous in natural environments and is recognized as one of the primary pathogens affecting farmed freshwater and marine fish worldwide (3). Infections caused by *E. tarda* typically result in a systemic condition known as edwardsiellosis, which is characterized by ascites, severe internal organ lesions, exophthalmia, and herniation (4). Since its first reported outbreak in 1962, edwardsiellosis has inflicted severe economic damage across more than 20 commercially important fish species, including tilapia (*Tilapia nilotica*), freshwater catfish (*Tandanus tandanus*), Japanese eel (*Anguilla japonica*), largemouth bass (*Micropterus salmoides*), rainbow trout (*Oncorhynchus mykiss*), turbot (*Scophthalmus maximus*), and mullet (*Mugil cephalus*) (5, 6). Currently, antibiotics remain the primary strategy for controlling *E. tarda* infections. However, increasing antibiotic resistance in *E. tarda*, particularly to cephalosporins, aminoglycosides, and penicillin, has significantly compromised treatment efficacy (7). Consequently, the development of novel, effective antimicrobial agents with anti-*E*. *tarda* activity is urgently needed.

Antimicrobial peptides (AMPs) are key effectors of the innate immune system, capable of mounting rapid and broad-spectrum defense responses against a wide range of pathogens, including bacteria, viruses, fungi, and parasites (8). AMPs can be naturally isolated from various organisms, obtained as peptide derivatives, or synthesized artificially. They are typically characterized by structural features such as hydrophobicity, amphiphilicity, and a net positive charge, although a small subset of AMPs is anionic. In addition, AMPs often adopt α-helical or β-sheet conformations (9). These properties enable AMPs to interact with anionic components on microbial membranes, leading to membrane disruption and leakage of intracellular contents. In some cases, AMPs can also exert intracellular effects, ultimately resulting in microbial death (10). Due to their multiple modes of action and rapid bactericidal activity, the likelihood of pathogens developing resistance to AMPs is considerably lower than to traditional antibiotics (11). These advantages position AMPs as promising alternatives to antibiotics in aquaculture.

To date, more than 3,300 AMPs have been identified across a wide range of organisms. However, fish-derived AMPs account for less than 5% of these peptides (Antimicrobial Peptide Database). Marine fish, due to their adaptation to environments characterized by high salinity, fluctuating temperatures, and high microbial pressure, represent a valuable reservoir for novel AMP discovery (12). The mudskipper *Boleophthalmus pectinirostris*, a unique amphibious teleost, inhabits estuarine and intertidal zones where it is exposed to complex environmental stressors. This ecological niche likely necessitates an enhanced reliance on innate immune effectors. To date, only five AMPs have been identified from *B. pectinirostris*, including BpLEAP-2 (13), BpHep-1 and BpHep-2 (14), BpNKL (15), and Bolespleenin_334-347_ (16). Among them, the first four belong to previously characterized AMP families, whereas Bolespleenin_334-347_ represents a newly discovered AMP with distinct structural features. Further exploration of novel AMPs from *B. pectinirostris* is essential to better understand its immune defense strategies and provide a foundation for the development of new antimicrobial agents.

In the present study, we identified a novel immune-related gene from *B. pectinirostris*, designated as *Pecbloodin*. The full-length cDNA of *Pecbloodin* was obtained using rapid amplification of cDNA-PCR (RACE-PCR), and its tissue expression profile was analyzed via quantitative real-time PCR (qPCR). A predicted AMP derived from this gene, named Pecbloodin_18-37_, was chemically synthesized based on CAMP_R4_ prediction, and its antimicrobial activity was evaluated. We investigated its mechanism of action, assessed its ability to circumvent bacterial resistance, and tested its efficacy using a *B. pectinirostris-E. tarda* infection model. The expression levels of immune-related genes (TNF-α, IL-1β, IL-10, and TLR4) and AMP-related genes (Hepcidin, LEAP-2, and lysozyme) in the liver were examined. Additionally, reactive oxygen species (ROS) levels and myeloperoxidase (MPO) activity were measured. These findings are expected to provide theoretical and practical insights into the development of novel anti-*E*. *tarda* agents with low risk of resistance development.

## RESULTS

### Sequence analysis of *Pecbloodin* and design of the truncated peptide

**Fig 1:**
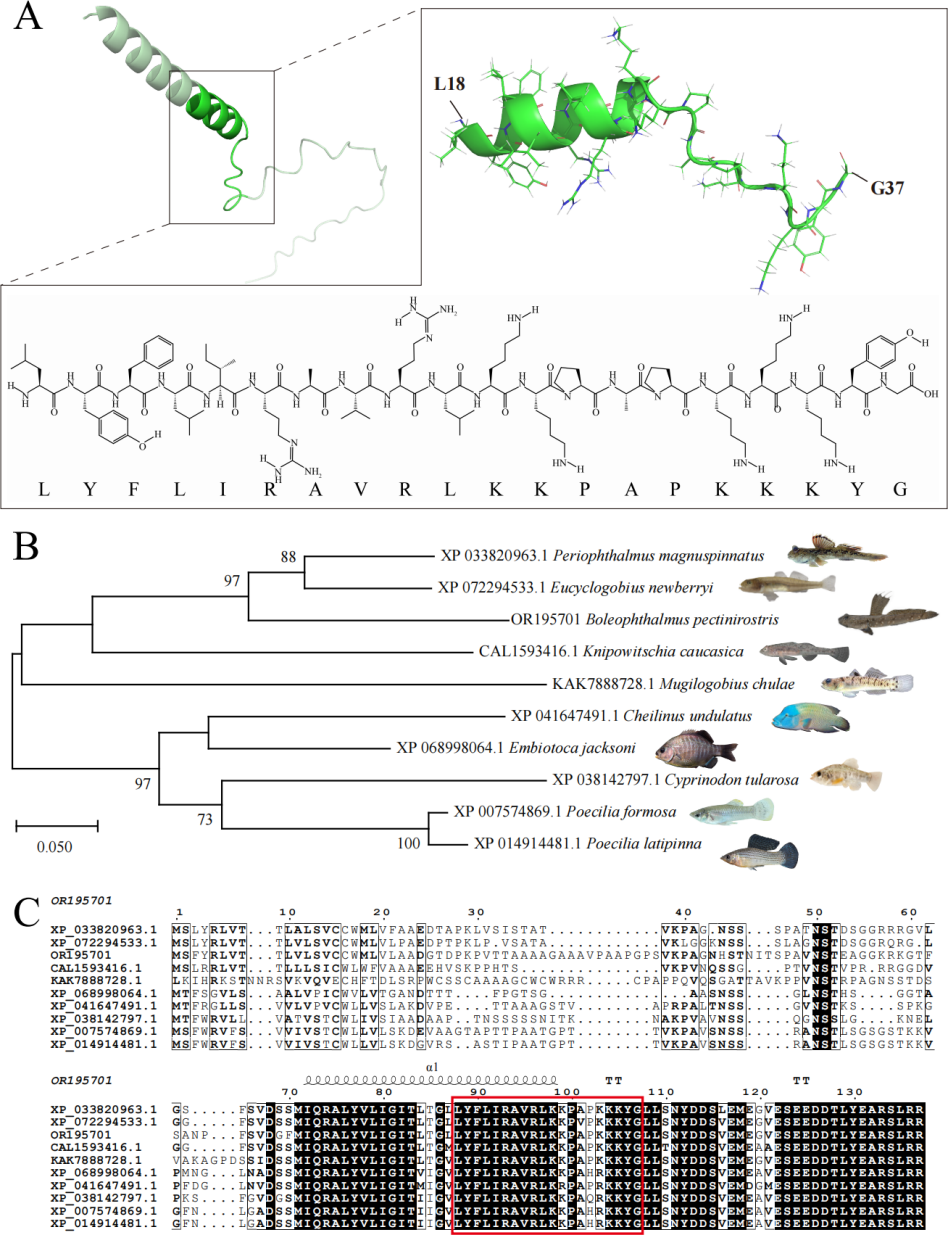
Structural analysis of *Pecbloodin* and Pecbloodin_18-37_, phylogenetic tree construction, and sequence alignment with homologs. (**A**) Predicted three-dimensional structures of *Pecbloodin* and its truncated peptide Pecbloodin_18-37_ generated by AlphaFold. (**B**) Phylogenetic tree of *Pecbloodin* and its homologs constructed using the neighbor-joining method in MEGA, with 1,000 bootstrap replicates. (**C**) Multiple sequence alignment of *Pecbloodin* with homologous proteins from other species. The amino acid sequence of Pecbloodin_18-37_ is highlighted in red within the boxed region.

The full-length cDNA sequence of *Pecbloodin* was successfully obtained and submitted to the GenBank database (accession No. OR195701). As shown in Fig. S1, the gene contains a 29-bp 5′ untranslated region (UTR) and a 366-bp 3′ UTR, with an open reading frame encoding a protein of 67 amino acids. The predicted molecular weight of the encoded protein is 7.75 kDa, and the theoretical isoelectric point (pI) is 7.98 (Table 1). Bioinformatic analysis identified a 20-residue peptide fragment within Pecbloodin, designated as Pecbloodin_18-37_ (H-LYFLIRAVRLKKPAPKKKYG-OH), with potential antimicrobial properties. As shown in Fig. 1A, the predicted tertiary structure of Pecbloodin_18-37_ adopts a typical α-helical conformation and contains seven basic amino acids, including lysine and arginine, which contribute to its cationic nature. Physicochemical property analysis revealed that Pecbloodin_18-37_ is an amphiphilic cationic peptide, with a net positive charge of +7 and a hydrophobicity of 40% (Table 1). Machine learning-based AMP prediction using the CAMP_R4_ platform indicated high confidence in its antimicrobial potential, with probability scores of 0.96 (Artificial Neural Network), 0.99 (Support Vector Machine), and 0.95 (Random Forest), collectively classifying Pecbloodin_18-37_ as an AMP.

**TABLE 1:** Sequence information and physicochemical properties of *Pecbloodin*, Pecbloodin_18-37_, and LL-37

Physicochemical parameter	*Pecbloodin*	Pecbloodin_18-37_	LL-37
Number of amino acids (aa)	67	20	37
Molecular weight (Da)	7,751.04	2,390.00	4,493.32
Theoretical pI	7.98	10.73	10.61
Molecular formula	C_349_H_570_N_92_O_102_S_2_	C_116_H_193_N_31_O_23_	C_205_H_340_N_60_O_53_
Total number of atoms	1,115	363	658
Grand average of hydropathicity	−0.219	−0.410	−0.724
Total hydrophobic ratio (%)	39	40	35
Total net charge	+1	+7	+6

Sequence alignment showed that the *Pecbloodin* protein shares 98.51% amino acid identity with two uncharacterized proteins from *Periophthalmus magnuspinnatus* (XP_033820963.1) and *Eucyclogobius newberryi* (XP_072294533.1). Phylogenetic analysis further confirmed that these homologous sequences cluster within the same evolutionary clade, and the truncated peptide region is highly conserved among them (Fig. 1B and C).

### Expression patterns of the *Pecbloodin* gene

**Fig 2:**
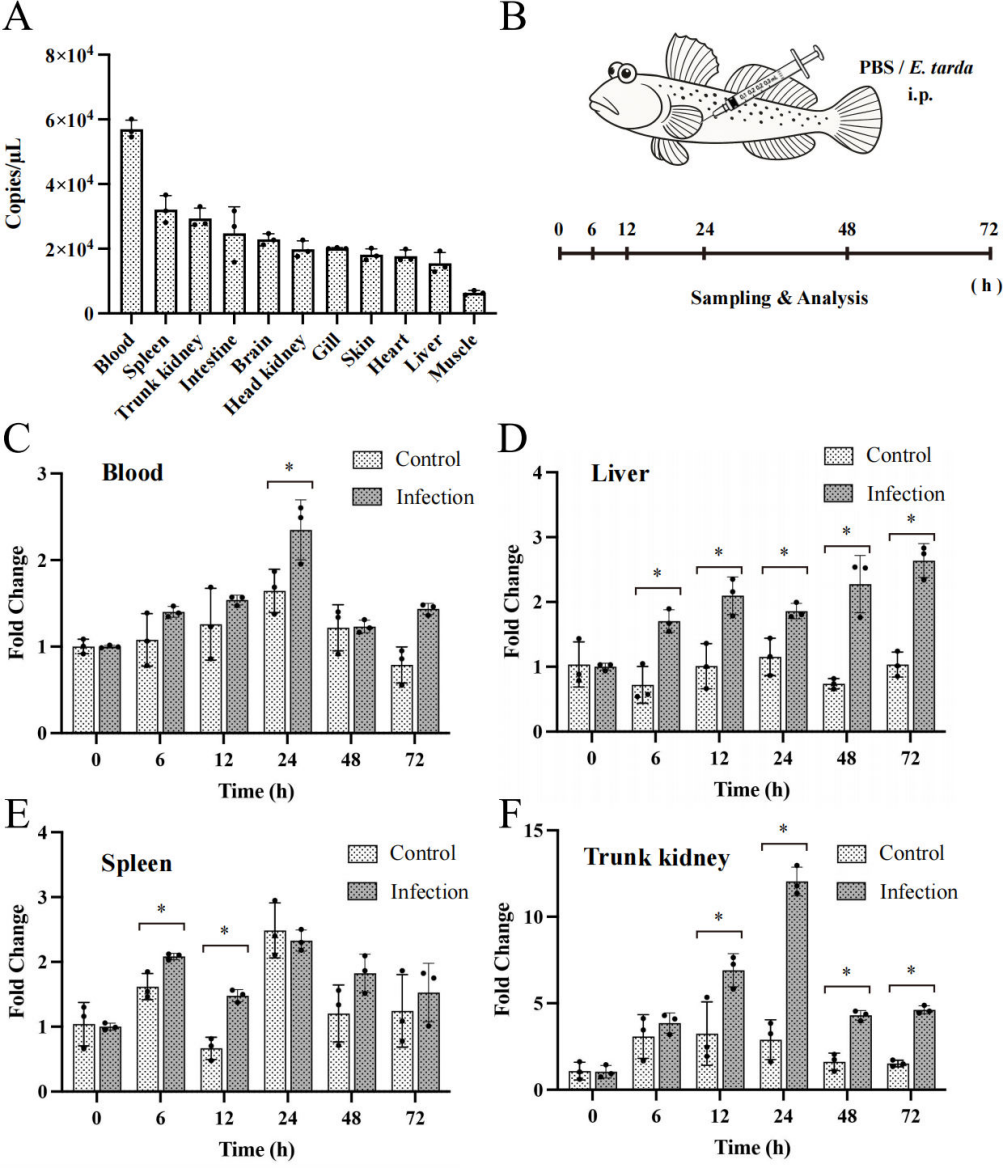
Tissue-specific expression and temporal regulation of *Pecbloodin* in *B. pectinirostris* following *E. tarda* infection. (**A**) Expression levels of *Pecbloodin* in various tissues under basal conditions (*n* = 3). (**B**) Schematic diagram of *E. tarda* infection and sampling timeline. (**C–F**) Temporal expression of *Pecbloodin* in blood, liver, spleen, and trunk kidney after infection (*n* = 3). Asterisks indicate statistically significant differences compared to the control group (*P < 0.05).

The tissue distribution of *Pecbloodin* expression in *B. pectinirostris* was analyzed by qPCR (Fig. 2A). The gene was found to be most highly expressed in blood, while the lowest expression level was observed in muscle.

To investigate the immune response of *Pecbloodin*, its expression was further assessed in *B. pectinirostris* following *E. tarda* infection (Fig. 2B). Upon infection, *Pecbloodin* expression was significantly upregulated in the blood at 24 h post-infection (hpi) (Fig. 2C), in the liver at 6, 12, 24, 48, and 72 hpi (Fig. 2D), in the spleen at 6 and 12 hpi (Fig. 2E), and in the trunk kidney at 12, 24, 48, and 72 hpi (Fig. 2F). These results suggest that *Pecbloodin* is responsive to *E. tarda* infection and may play a role in the innate immune defense of multiple tissues.

### Antimicrobial activity of Pecbloodin_18-37_

The antimicrobial activity of Pecbloodin_18-37_ was evaluated against a panel of microorganisms, as summarized in Table 2. The peptide exhibited broad-spectrum antibacterial activity. It effectively inhibited the growth of various gram-negative bacteria, including *Pseudomonas aeruginosa*, *Acinetobacter baumannii*, and *Vibrio alginolyticus*, as well as gram-positive bacteria, such as *Listeria monocytogenes*, *Staphylococcus aureus*, *Corynebacterium glutamicum*, *Enterococcus faecalis*, and *Bacillus cereus*, with minimum inhibitory concentration (MIC) values ≤6  µM and minimum bactericidal concentration (MBC) values ≤12  µM.

**TABLE 2:** Broad-spectrum antimicrobial assay for Pecbloodin_18-37_

Strain	Pecbloodin_18-37_			LL-37 MIC (μM)
	CGMCC no.^*a*^	MIC^*b*^ (μM)	MBC^*b*^/MFC^*b*^ (μM)	
Gram-negative bacteria				
*Escherichia coli*	1.2385	24–48	24–48	12–24
*Pseudomonas aeruginosa*	1.2421	1.5–3	3–6	6–12
*Acinetobacter baumannii*	1.6769	1.5–3	1.5–3	3–6
*Edwardsiella tarda*	1.1872	12–24	24–48	48–96
*Aeromonas hydrophila*	1.2017	12–24	24–48	48–96
*Vibrio alginolyticus*	1.1833	3–6	6–12	12–24
Gram-positive bacteria				
*Listeria monocytogenes*	1.10753	1.5–3	1.5–3	1.5–3
*Staphylococcus epidermidis*	1.4260	12–24	12–24	3–6
*Staphylococcus aureus*	1.2465	3–6	3–6	6–12
*Corynebacterium glutamicum*	1.1886	<1.5	1.5–3	1.5–3
*Enterococcus faecalis*	1.2135	3–6	3–6	6–12
*Bacillus cereus*	1.3760	3–6	3–6	<1.5
Fungi				
*Cryptococcus neoformans*	2.1563	<1.5	<1.5	1.5–3
*Candida albicans*	2.2411	3–6	6–12	6–12
*Aspergillus flavus*	3.4410	24–48	48–96	12–24
*Fusarium oxysporum*	3.6785	12–24	12–24	12–24
*Fusarium solani*	3.5840	6–12	6–12	6–12
Multidrug-resistant strains^*c*^				
MRSA-QZ19130	–^*d*^	3–6	6–12	12–24
MRSA-QZ19134	–	3–6	3–6	12–24
*P. aeruginosa*-QZ19121	–	3–6	6–12	6–12
*P. aeruginosa*-QZ19122	–	1.5–3	3–6	6–12
*A. baumannii*-QZ18050	–	1.5–3	1.5–3	3–6
*A. baumannii*-QZ18055	–	3–6	3–6	6–12

^*a*^ Strain accession numbers correspond to entries in the China General Microbiological Culture Collection Center.

^*b*^ Antimicrobial activity is reported in the A–B format, where A represents the highest concentration at which visible microbial growth was still observed, and B indicates the lowest concentration at which no visible growth occurred.

^*c*^ Multidrug-resistant clinical isolates were obtained from the Second Affiliated Hospital of Fujian Medical University (Quanzhou, Fujian, China).

^*d*^ – indicates not applicable; these strains are clinical isolates and therefore have no corresponding CGMCC number.

In addition to its antibacterial effects, Pecbloodin_18-37_ demonstrated antifungal activity against both yeast-like fungi and filamentous fungi, including *Cryptococcus neoformans*, *Candida albicans*, *Fusarium oxysporum*, and *Fusarium solani*, with MIC values ≤ 12  µM and MBC values ≤ 24  µM. The peptide also inhibited the conidial germination of filamentous fungi, such as *F. solani*, *Fusarium graminearum*, *Aspergillus flavus*, and *F. oxysporum*, as illustrated in Fig. S2.

Furthermore, the efficacy of Pecbloodin_18-37_ was assessed against clinically isolated multidrug-resistant (MDR) strains. Remarkably, the peptide showed potent inhibitory effects against MRSA (QZ19134), MDR *P. aeruginosa* (QZ19122), and MDR *A. baumannii* (QZ18050 and QZ18055), with MIC values as low as 6 μM. These findings suggest that Pecbloodin_18-37_ may serve as a promising candidate for combating antibiotic-resistant pathogens.

### Bactericidal kinetics and stability of Pecbloodin_18-37_

**Fig 3:**
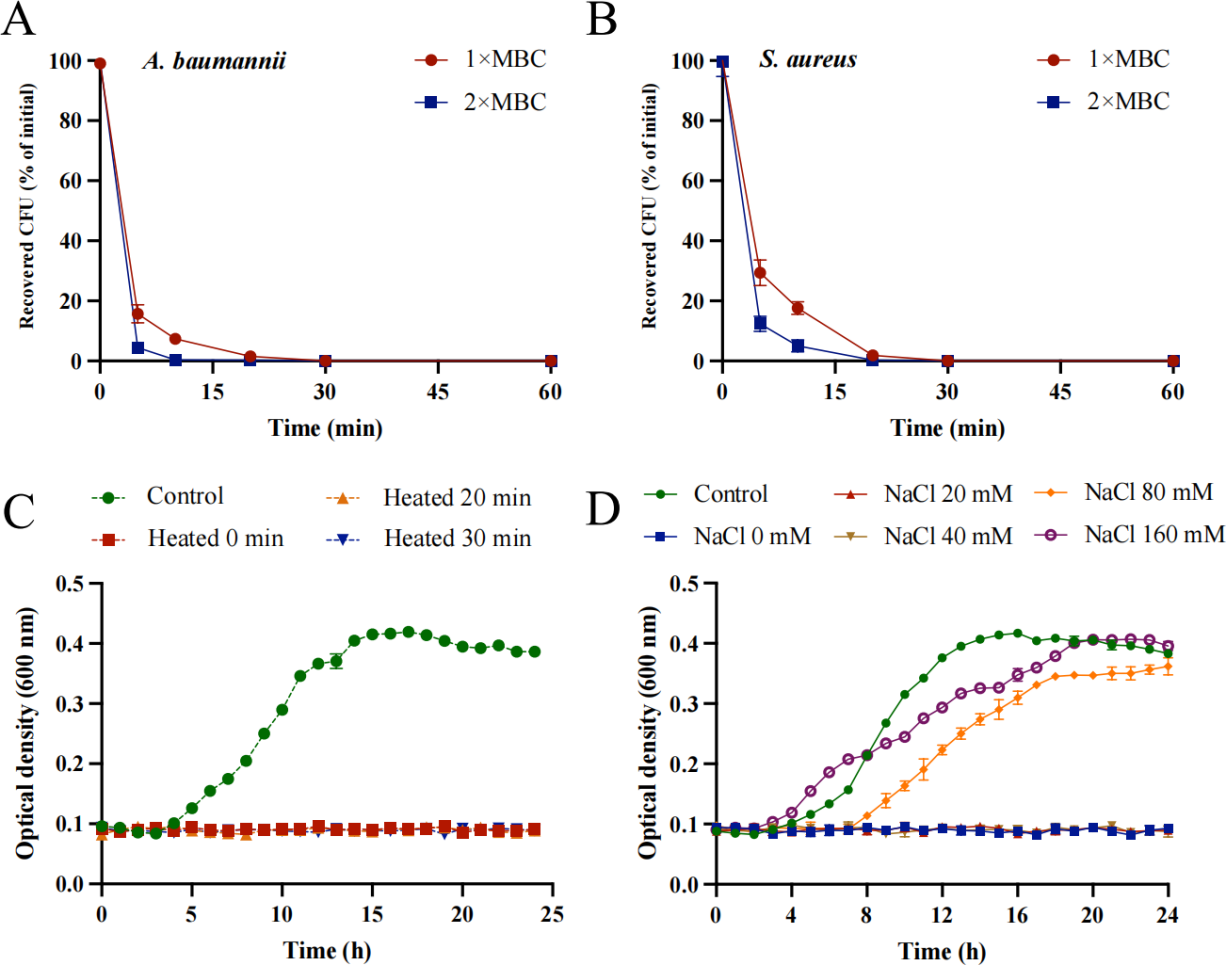
Bactericidal kinetics and stability of Pecbloodin_18-37_. (**A and B**) Time-dependent killing curves of Pecbloodin_18-37_ against *A. baumannii* and *S. aureus* at 1× and 2× MBC. (**C**) Thermal stability of Pecbloodin_18-37_ after treatment at 100°C for up to 30 min. (**D**) Antibacterial activity of Pecbloodin_18-37_ against *A. baumannii* under varying sodium ion concentrations, assessed by OD_595_. All data are expressed as mean ± SEM from three biological replicates.

Time-kill kinetic assays demonstrated the rapid bactericidal activity of Pecbloodin_18-37_. At 1× MBC, the peptide reduced the viability of both *A. baumannii* and *S. aureus* by over 99% within 30 min. At a higher concentration (2× MBC), this >99% reduction was achieved even faster—within 10 min for *A. baumannii* (Fig. 3A) and 20 min for *S. aureus* (Fig. 3B).

The stability of Pecbloodin_18-37_ under thermal and ionic stress conditions was also evaluated. Thermal stability testing demonstrated that the peptide retained its antimicrobial activity against *A. baumannii* even after heat treatment at 100°C for up to 30 min (Fig. 3C), indicating strong heat resistance. Additionally, the effect of different sodium ion concentrations on its activity was examined. The results showed that Pecbloodin_18-37_ could not completely inhibit the growth of *S. aureus* at a sodium ion concentration of 80 mM (Fig. 3D).

### Induction of endogenous ROS in bacteria by Pecbloodin_18-37_

AMPs are known to promote bacterial cell damage in part by inducing the accumulation of ROS (17). To assess whether Pecbloodin_18-37_ exerts similar effects, intracellular ROS levels were measured in *A. baumannii* and *S. aureus* following peptide treatment.

**Fig 4:**
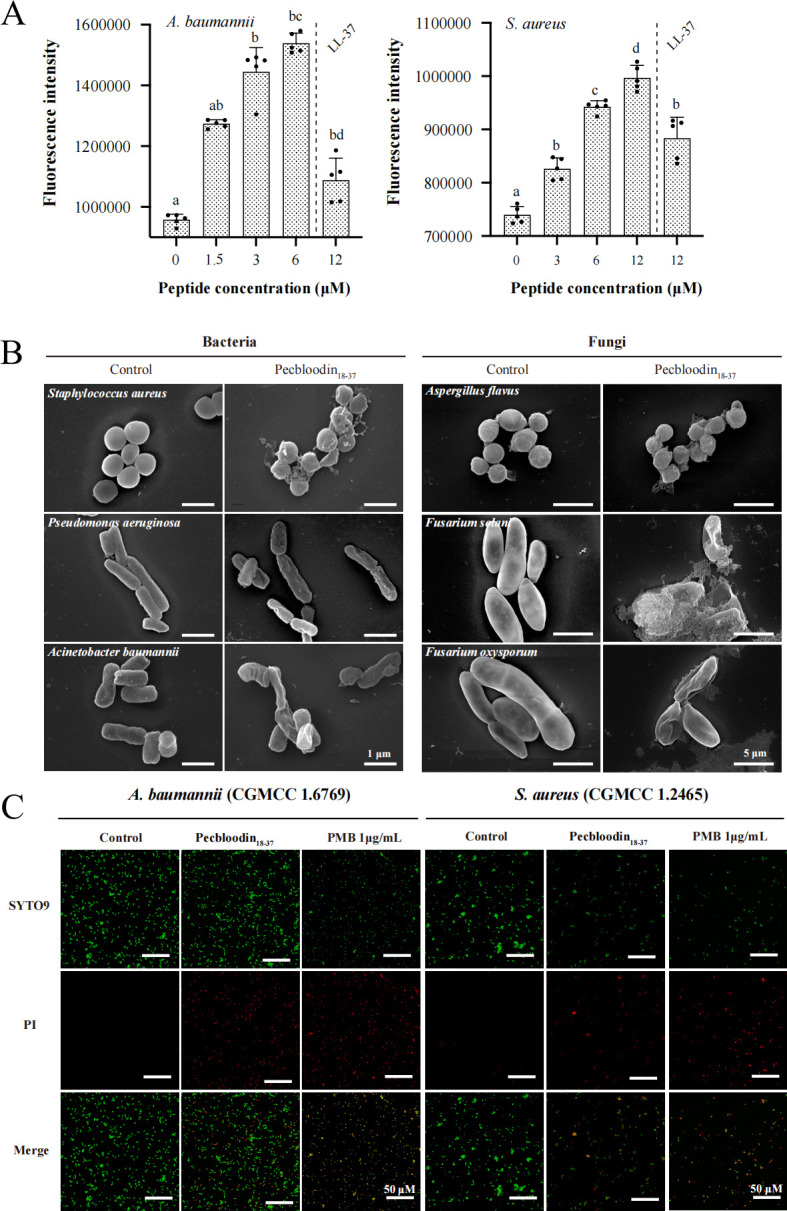
Effects of Pecbloodin_18-37_ on ROS generation and microbial membrane integrity. (**A**) Intracellular ROS production in *A. baumannii* and *S. aureus* following Pecbloodin_18-37_ treatment, detected via DCFH-DA fluorescence (*n* = 5). Different lowercase letters denote statistically significant differences among groups (*P* < 0.05). (**B**) Scanning electron microscopy images of bacterial and fungal cells showing morphological changes after treatment with Pecbloodin_18-37_. (**C**) CLSM analysis of membrane permeability in *A. baumannii* and *S. aureus* stained with SYTO 9 and PI.

As shown in Fig. 4A, Pecbloodin_18-37_ significantly increased ROS production in both bacterial species in a dose-dependent manner. Higher peptide concentrations resulted in greater accumulation of endogenous ROS, suggesting that ROS generation may contribute to the bactericidal mechanism of Pecbloodin_18-37_.

### Morphological alterations in microorganisms induced by Pecbloodin_18-37_

Scanning electron microscopy (SEM) was employed to examine the morphological changes in bacterial (*S. aureus*, *P. aeruginosa*, and *A. baumannii*) and fungal (*A. flavus*, *F. solani*, and *F. oxysporum*) cells following treatment with Pecbloodin_18-37_.

As shown in Fig. 4B, treatment with Pecbloodin_18-37_ resulted in severe structural damage to both bacterial and fungal cells, including membrane disruption, surface collapse, and leakage of intracellular contents. In contrast, untreated cells displayed smooth, intact surfaces with no observable damage, indicating that Pecbloodin_18-37_ directly compromises microbial membrane integrity.

### Pecbloodin_18-37_ increases bacterial inner membrane permeability

The effect of Pecbloodin_18-37_ on the inner membrane integrity of *A. baumannii* and *S. aureus* was evaluated using SYTO 9 and propidium iodide (PI) fluorescence staining. SYTO 9 stains all bacterial cells, while PI penetrates only those with compromised membranes, emitting red fluorescence.

As shown in Fig. 4C, untreated bacteria displayed uniform green fluorescence, indicating intact membranes. In contrast, Pecbloodin_18-37_-treated bacteria exhibited strong red fluorescence, comparable to that observed in the polymyxin B (PMB)-treated group, suggesting significant disruption of inner membrane integrity.

### Inhibitory effect of Pecbloodin_18-37_ on biofilm formation

Biofilm formation is a common feature of bacterial infections and is a major contributor to antibiotic resistance (18), potentially increasing bacterial tolerance by 10- to 1,000-fold (19). To evaluate the anti-biofilm potential of Pecbloodin_18-37_, varying concentrations (6–48 μM) were tested against *A. baumannii* and *S. aureus*.

**Fig 5:**
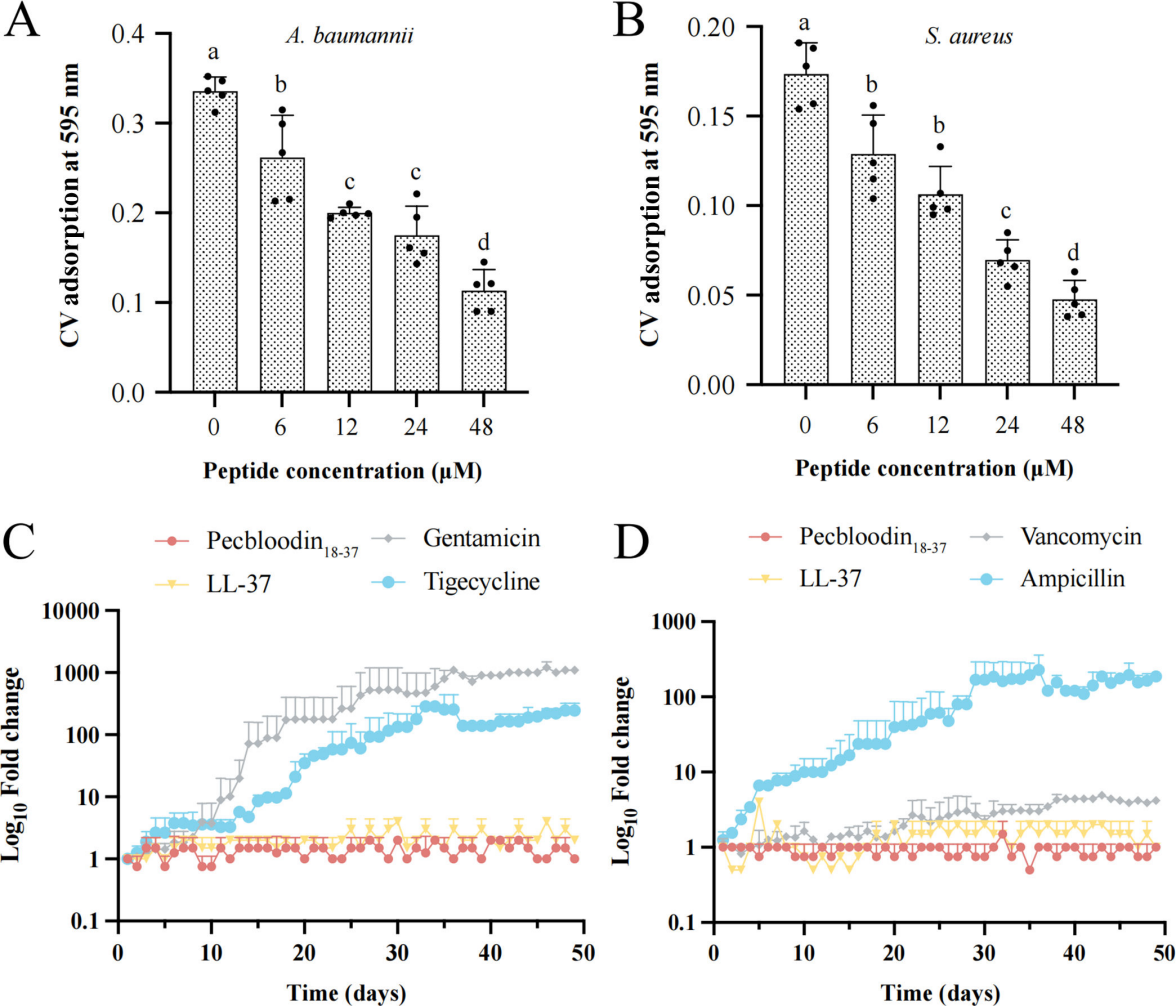
Pecbloodin_18-37_ inhibits biofilm formation and does not induce bacterial resistance. (**A and B**) Quantification of biofilm formation by *A. baumannii* and *S. aureus* treated with varying concentrations of Pecbloodin_18-37_ using crystal violet staining and OD_595_ measurements (*n* = 5). Data are shown as mean ± SEM. Statistical significance was assessed using one-way ANOVA followed by the Dunnett test; different letters indicate significant differences. (**C and D**) Resistance induction assay over 48 days under sub-MIC exposure. MIC fold change is plotted on a log10 scale against time. LL-37 and antibiotics served as controls.

As shown in Fig. 5A and B, Pecbloodin_18-37_ significantly inhibited biofilm formation in both bacterial species in a dose-dependent manner, suggesting its potential utility in disrupting biofilm-associated resistance mechanisms.

### Pecbloodin_18-37_ exhibits antibacterial activity without inducing resistance

The rapid emergence of antibiotic resistance in clinical pathogens, particularly *A. baumannii* and *S. aureus*, poses a significant challenge to effective treatment (20). To assess the risk of resistance development, both bacterial species were subjected to 48 consecutive days of exposure to sub-MIC concentrations of two AMPs (Pecbloodin_18-37_ and LL-37) and four conventional antibiotics.

In *A. baumannii*, prolonged exposure to Pecbloodin_18-37_ and LL-37 did not result in any notable change in MIC values. In contrast, gentamicin resistance increased by nearly 1,000-fold, while tigecycline resistance increased by several hundred fold (Fig. 5C). Similarly, *S. aureus* showed no significant change in susceptibility to either AMP. However, the MIC for ampicillin increased by several hundred fold and that for vancomycin increased by less than 10-fold. Despite the relatively modest change, the increase in vancomycin resistance was statistically significant and may indicate a concerning trend (Fig. 5D). These findings underscore the low propensity of Pecbloodin_18-37_ to induce resistance compared to conventional antibiotics, highlighting its potential as a promising antimicrobial agent for long-term application.

### Pecbloodin_18-37_ exhibits low cytotoxicity and hemolytic activity

**Fig 6:**
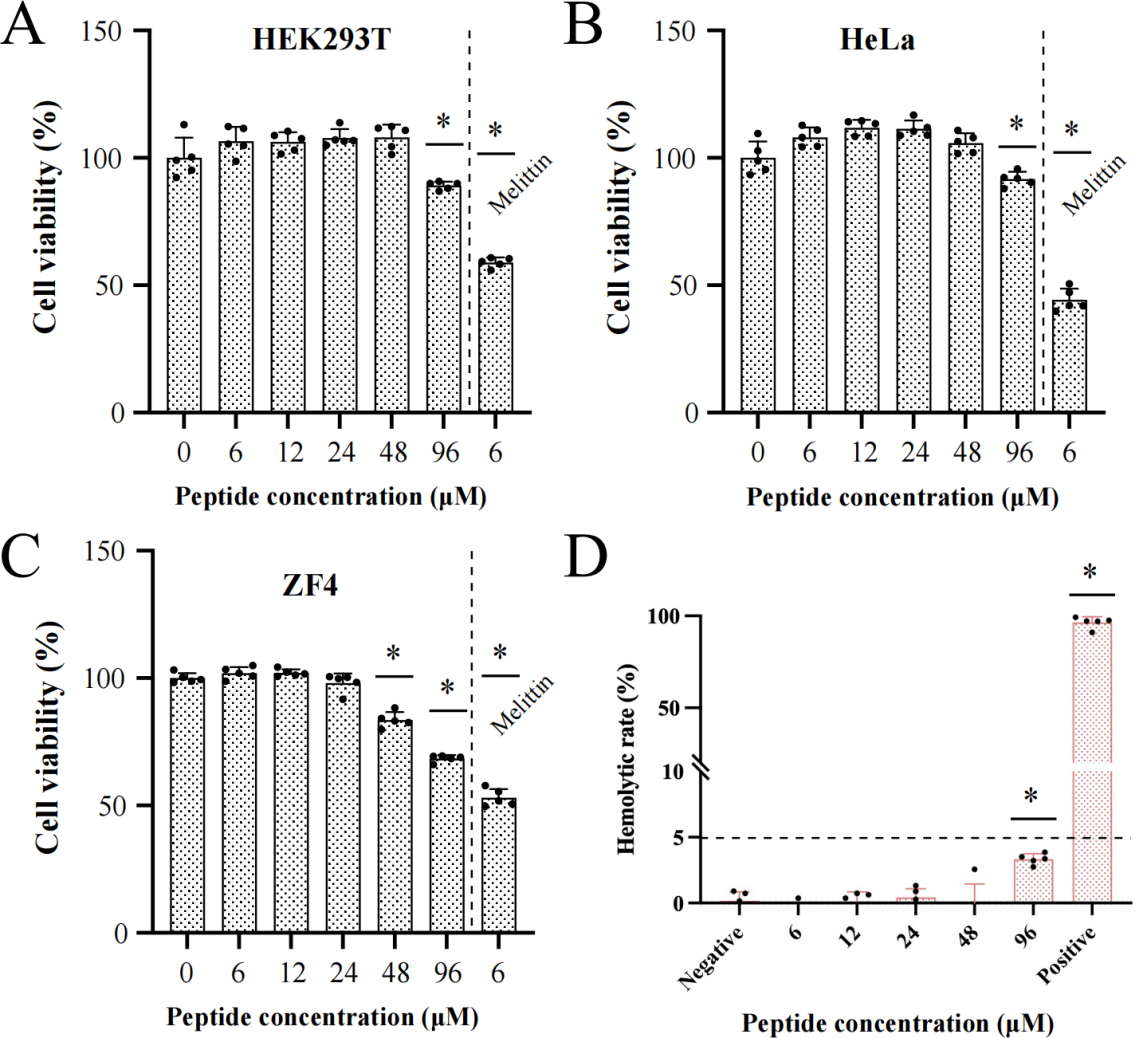
*In vitro* cytotoxicity and hemolytic activity of Pecbloodin_18-37_. (**A–C**) Cell viability of HEK-293T, HeLa, and ZF4 cells after exposure to Pecbloodin_18-37_, measured by MTS assay. Melittin served as a cytotoxic control. (**D**) Hemolytic activity of Pecbloodin_18-37_ on mouse erythrocytes. Saline and 0.1% Triton X-100 were used as negative and positive controls, respectively. Data are presented as mean ± SEM. *P < 0.05 indicates a statistically significant difference.

The cytotoxicity of Pecbloodin_18-37_ was evaluated using two normal cell lines (HEK-293T and ZF4) and one cancer cell line (HeLa). As shown in Fig. 6A through C, Pecbloodin_18-37_ exhibited no significant cytotoxicity toward HEK-293T and HeLa cells at concentrations below 96 μM and no cytotoxic effects on ZF4 cells at concentrations below 48 μM. In contrast, Melittin (6 μM), used as a positive control, induced marked cytotoxicity in all three cell lines.

The hemolytic activity of Pecbloodin_18-37_ was assessed using freshly isolated mouse erythrocytes. Saline (0.9%) served as a negative control and caused no hemolysis, while 0.1% Triton X-100, used as a positive control, resulted in complete lysis of red blood cells. As shown in Fig. 6D, Pecbloodin_18-37_ caused no significant hemolysis at concentrations below 96 μM, indicating favorable hemocompatibility.

### *In vivo* efficacy of Pecbloodin_18-37_ in an *E. tarda*-infected *B. pectinirostris* model

An *in vivo* infection model of *B. pectinirostris* was established to evaluate the antimicrobial, immunomodulatory, and antioxidant effects of Pecbloodin_18-37_. Fish were intraperitoneally injected with *E. tarda*, followed by administration of Pecbloodin_18-37_ 1 h post-infection.

**Fig 7:**
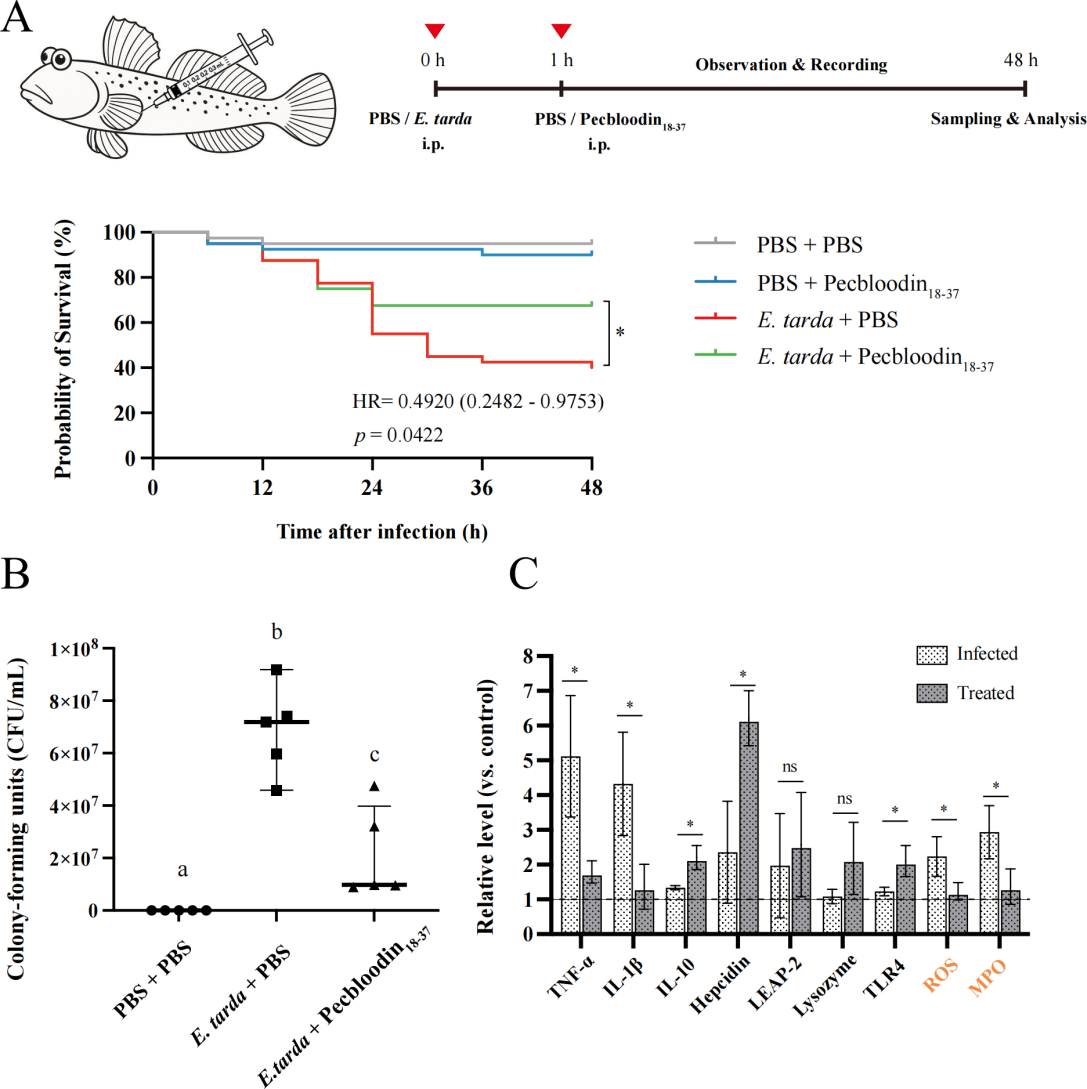
*In vivo* immunoprotective effects of Pecbloodin_18-37_ against *E. tarda* infection in *B. pectinirostris.* (**A**) Experimental scheme and Kaplan-Meier survival curves for mudskippers infected with *E. tarda* and treated with Pecbloodin_18-37_ (*n* = 40). (**B**) Liver bacterial load after treatment (*n* = 5). (**C**) Expression levels of immune-related genes (TNFα, IL-1β, IL-10, TLR4, and Hepcidin), ROS levels, and MPO activity in liver tissue (*n* = 5). Statistical comparisons were conducted using one-way ANOVA or *t*-tests as appropriate. Different letters or asterisks denote significant differences (*P < 0.05).

At 48 h post-treatment, the survival rate in the Pecbloodin_18-37_-treated group was 67.5%, significantly higher than the 42.5% observed in the control group (*P* = 0.0422). The hazard ratio for the treatment group relative to the control was 0.4920 (95% CI: 0.2482–0.9753), indicating that Pecbloodin_18-37_ significantly reduced mortality risk (Fig. 7A).

Bacterial burden analysis showed a marked reduction in liver CFU counts in the treated group compared to the control (Fig. 7B). qPCR analysis of liver tissue revealed significant downregulation of pro-inflammatory cytokines (TNF-α and IL-1β) and upregulation of the anti-inflammatory cytokine IL-10, the antimicrobial peptide gene Hepcidin, and the pattern recognition receptor TLR4 in the Pecbloodin_18-37_-treated group. Moreover, Pecbloodin_18-37_ treatment significantly decreased hepatic ROS levels and MPO activity, indicating alleviation of infection-associated oxidative stress (Fig. 7C).

## DISCUSSION

Intensive aquaculture practices have resulted in the widespread occurrence of various diseases, especially edwardsiellosis, which is notable for its high incidence rate (21). To combat bacterial infections in aquaculture, a significant quantity of antibiotics is used (22). Nevertheless, the rise of antibiotic resistance in aquaculture and its implications for public health and food safety have underscored the necessity for developing novel antibacterial drugs. The marine environment is a rich reservoir of bioactive molecules, where various immune-related substances play crucial roles in pathogen recognition and clearance (23–25). AMPs are regarded as a highly viable substitute for traditional antibiotics. They possess broad-spectrum antibacterial activity, fast bactericidal action, low potential for drug resistance, and are characterized by their safety, minimal toxicity, and lack of side effects (26). Mudskippers *B. pectinirostris*, which are amphibious fish inhabiting the tidal flats, have a series of unique physiological traits that enable them to thrive in the complex environment straddling land and sea (27). Owing to this intricate habitat, mudskippers are constantly exposed to various disease-causing microbes. Therefore, there is great potential to mine novel and effective antimicrobial peptides from this species. In our research, we discovered a novel and previously uncharacterized functional gene, *Pecbloodin*, from *B. pectinirostris*. Following bioinformatics examination and confirmation, a truncated peptide named Pecbloodin_18-37_ was identified from *Pecbloodin*. We investigated the physicochemical characteristics and antimicrobial activity of Pecbloodin_18-37_ to understand its antimicrobial efficacy against *E. tarda* infections and its underlying mechanism, offering new perspectives for developing antibiotic substitutes for future aquaculture applications.

Due to the immature adaptive immunity of fish, they primarily rely on their innate immune system to resist pathogenic microorganisms when faced with infection (28). The primary immune organs involved include the intestine, liver, spleen, and kidney (29). In this study, *Pecbloodin* was significantly upregulated in the blood, liver, spleen, and trunk kidney upon *E. tarda* challenge. These findings indicate that *Pecbloodin* may play a crucial role in the immune protection of mudskippers. Currently, it is commonly acknowledged that the net charge and hydrophobicity of AMPs are among the most significant factors influencing their antimicrobial activity (30). Pecbloodin_18-37_ possesses a substantial positive charge number of +7 and 40% hydrophobicity, similar to LL-37, and exhibits potent broad-spectrum antimicrobial efficacy against various bacterial and fungal pathogens. Similar to the binding properties of other cationic AMPs (31), the positive charge of Pecbloodin_18-37_ might enhance binding to negatively charged microbial surfaces through electrostatic interaction.

One of the bactericidal mechanisms of AMPs is the disruption of bacterial membrane structure, which typically relies on the peptide concentration at the membrane surface. Once a specific threshold is reached, the membrane structure is destroyed (32, 33). In this study, we examined the impact of Pecbloodin_18-37_ on the morphological structure of bacteria using SEM and verified its effect on bacterial membrane permeability through SYTO9 and PI staining. Pecbloodin_18-37_ was shown to interact with cell membranes, causing them to rupture and shrink on the surface, which leads to leakage of cellular contents and ultimately bacterial death. Furthermore, we found that the endogenous ROS in bacteria significantly rose after exposure to Pecbloodin_18-37_. This indicates that Pecbloodin_18-37_ simultaneously induces oxidative stress, and elevated levels of ROS may injure bacterial lipids, proteins, and DNA, leading to lethal bacterial damage (34). In conclusion, the disruption of membrane integrity and the surge in ROS levels may both contribute to bacterial killing.

Biofilms serve as a means for bacteria to withstand environmental stress and counteract the impact of drugs, including antibiotics (35). The development of bacterial biofilms generally includes three phases: (i) the planktonic stage, (ii) initial adherence to a surface, and (iii) the development of microcolonies and the release of extracellular polymeric substances (EPSs). When a thick EPS matrix is established, it restricts drug penetration, rendering it challenging to attain effective bactericidal concentrations within the biofilm, thereby conferring resistance (36). In this study, the bactericidal kinetics of Pecbloodin_18-37_ showed rapid killing of planktonic bacteria, thereby inhibiting further biofilm development. Moreover, at long-term sub-MIC concentrations, Pecbloodin_18-37_ did not induce resistance, which may be attributed to the multiple antimicrobial mechanisms of AMPs that limit bacterial resistance due to target mutations. This represents a significant edge of Pecbloodin_18-37_ over traditional antibiotics in preventing bacterial resistance.

Although AMPs have attracted widespread interest due to their myriad of advantages, the clinical application of AMPs is confronted with major obstacles, including poor stability, cytotoxicity, and production costs (10). Generally speaking, the activity of AMPs is notably influenced by elevated levels of sodium ions (37). The toxicity of certain cationic peptides also significantly limits their applications. For instance, Melittin, which possesses bactericidal properties, exhibits marked toxicity toward mammalian cells, although this property has also been harnessed for cancer studies (38). This study further evaluates the stability and safety of Pecbloodin_18-37_, with results indicating that high-temperature treatment has no significant impact on its activity. Moreover, the antibacterial activity of Pecbloodin_18-37_ remained largely unaffected when the sodium concentration was 40 mM, and although there was some impact at 80 mM sodium concentration, it can still inhibit bacterial growth within 7 h, demonstrating that Pecbloodin_18-37_ has a relatively high tolerance to sodium ions. In terms of safety assessment, while Pecbloodin_18-37_ exhibits cytotoxic effects on HEK-293T and HeLa cells at a concentration of 96 μM and on ZF4 cells at 48 μM, in comparison, the positive peptide control Melittin exhibits notable toxicity even at a low concentration of 6 μM. Additionally, the bactericidal concentrations of Pecbloodin_18-37_ for most bacteria or fungi are below 24 μM, rendering it relatively safe for practical applications. Subsequently, the hemolytic capacity of Pecbloodin_18-37_ in mouse red blood cells was further assessed, and no marked hemolysis was detected at a concentration of 48 μM. It is a well-established fact that cell membranes are enriched in neutral phospholipids like phosphatidylcholine and sphingomyelin, in contrast to bacterial membranes. The bulk of negatively charged phospholipids, such as phosphatidylserine, are predominantly situated in the inner layer of the bilayer (37). Thus, the selectivity of Pecbloodin_18-37_ might be significantly influenced by the variations in membrane composition and structure. In summary, Pecbloodin_18-37_ demonstrates excellent stability and biocompatibility, holding promising prospects for future applications.

The *in vivo* anti-infection efficacy evaluation of antimicrobial peptides is a crucial step toward their practical application. Prior research has demonstrated that AMPs have both direct antimicrobial properties and a substantial impact on modulating the immune response *in vivo*, which aids in controlling microbial infections in organisms (39). AMPs derived from the mudskipper, such as BpNKL (15), BpHep-2 (40), and BpLEAP-2 (13), have been shown to significantly boost the survival rate of mudskippers infected with *E. tarda* and to markedly lower the bacterial load in tissues. Additionally, BpLEAP-2 could also decrease the mRNA expression levels of pro-inflammatory factors in tissues. In this study, we found that Pecbloodin_18-37_ similarly enhanced the survival rate of mudskippers infected with *E. tarda* and effectively reduced the bacterial load in liver tissues. Meanwhile, Pecbloodin_18-37_ could significantly decrease the mRNA expression levels of pro-inflammatory factors TNF-α and IL-1β and increase the expression of IL-10, TLR4, and Hepcidin. As an anti-inflammatory agent, IL-10 can significantly diminish the synthesis of pro-inflammatory factors and holds a crucial position in the treatment of inflammatory and autoimmune diseases (41). The activation of TLR4 can bind to lipopolysaccharides in gram-negative bacteria and, together with the highly expressed AMPs such as Hepcidin, accelerate bacterial killing (42). In addition, Pecbloodin_18-37_ also reduces the levels of ROS and the enzyme activity of MPO in liver tissues. The decrease in MPO activity reflects the reduction in ROS levels, and reactive oxygen species are considered harmful to tissues through oxidative DNA damage to cells (43). In summary, Pecbloodin_18-37_ can not only directly kill bacteria in mudskippers but also inhibit *E. tarda* infection in mudskippers through immune regulation and antioxidant effects.

In this study, a novel functional gene, *Pecbloodin*, was identified from the mudskipper *B. pectinirostris*. Based on its physicochemical characteristics, a truncated antimicrobial peptide, Pecbloodin_18-37_, was screened and found to exhibit broad-spectrum antimicrobial activity and excellent thermal stability. Pecbloodin_18-37_ exerts its bactericidal effect by disrupting microbial membrane integrity, increasing membrane permeability, and inducing the accumulation of ROS, ultimately leading to cell death. In addition, Pecbloodin_18-37_ effectively inhibits biofilm formation and shows a low risk of inducing bacterial resistance. *In vivo* assays further demonstrated that Pecbloodin_18-37_ significantly improves the survival of *E. tarda*-infected mudskippers, reduces hepatic bacterial burden, and modulates immune and oxidative stress responses. Overall, Pecbloodin_18-37_ represents a promising antimicrobial agent with significant potential for therapeutic application in aquaculture, offering a viable alternative to conventional antibiotics.

## MATERIALS AND METHODS

### Animals, strains, and cell lines

Mudskippers (*B. pectinirostris*, 20 ± 5 g) were obtained from a local aquaculture facility in Xiapu, Fujian, China. Prior to experimentation, fish were acclimated for at least 7 days in a recirculating aquaculture system maintained at 25°C with 10‰ salinity. Fish were anesthetized using 200 mg/L ethyl 3-aminobenzoate methanesulfonate (MS-222; Sigma-Aldrich, USA) before injection or tissue sampling.

Standard microbial strains used in this study were acquired from the China General Microbiological Culture Collection Center (CGMCC), including *E. coli* (CGMCC 1.2389), *P. aeruginosa* (CGMCC 1.2421), *A. baumannii* (CGMCC 1.6769), *E. tarda* (CGMCC 1.1872), *A. hydrophila* (CGMCC 1.2017), *V. alginolyticus* (CGMCC 1.1833), *L. monocytogenes* (CGMCC 1.10753), *S. epidermidis* (CGMCC 1.4260), *S. aureus* (CGMCC 1.2465), *C. glutamicum* (CGMCC 1.1886), *E. faecalis* (CGMCC 1.2135), *B. cereus* (CGMCC 1.3760), *C. neoformans* (CGMCC 2.1563), *C. albicans* (CGMCC 2.2411), *A. flavus* (CGMCC 3.4410), *F. oxysporum* (CGMCC 3.6785), *F. graminearum* (CGMCC 3.349), and *F. solani* (CGMCC 3.5840). Clinically relevant multidrug-resistant strains, including MRSA QZ19130/QZ19134, MDR *P. aeruginosa* QZ19121/QZ19122, and MDR *A. baumannii* QZ18050/QZ18055, were kindly provided by the Second Affiliated Hospital of Fujian Medical University.

HEK-293T and HeLa cell lines were obtained from the Chinese Academy of Sciences Cell Bank and maintained in DMEM (Gibco, USA) supplemented with 10% fetal bovine serum (FBS; Gibco, USA) at 37°C in a 5% CO_2_ incubator. The ZF4 cell line was purchased from the China Zebrafish Resource Center (Wuhan, China) and cultured in DMEM/F-12 (1:1) medium (Gibco, USA) with 10% FBS at 28°C under 5% CO_2_. Fresh mouse red blood cells were collected for hemolysis assays.

### Cloning of the *Pecbloodin* gene

Specific primers were designed using Primer Premier 5.0 (Premier Biosoft) and synthesized by Sangon Biotech (Shanghai, China). Total RNA was extracted from various tissues using TRIzol Reagent (Invitrogen, UK) following the manufacturer’s protocol. RNA concentration and purity were evaluated with an Agilent 2100 Bioanalyzer (Agilent Technologies, USA), and integrity was confirmed by 1.0% agarose gel electrophoresis. Equal quantities of RNA from different tissues were pooled for downstream cDNA synthesis. First-strand cDNA was synthesized using the SMARTer RACE 5′/3′ Kit (Clontech, USA), and full-length cDNAs were amplified using LA Taq DNA polymerase (Takara, Japan), a high-fidelity enzyme optimized for long-fragment amplification (44). The resulting PCR products were ligated into the pMD18-T vector (Takara, Japan), transformed into *E. coli*, and subjected to bidirectional Sanger sequencing by Sangon Biotech Co., Ltd. (Shanghai, China).

### Bioinformatic analysis, peptide design, and synthesis

Sequence alignment was conducted using DNAMAN 8.0. The 3D structure of Pecbloodin was predicted with AlphaFold (https://github.com/deepmind/alphafold) and visualized using PyMOL 2.5.8. Peptide chemical structures were drawn using ChemDraw (https://www.perkinelmer.com/category/chemdraw). Conserved domain analysis was performed via NCBI tools (NCBI; http://www.ncbi.nlm.nih.gov), and phylogenetic trees were constructed using MEGA 11.0. Sequence alignments were visualized with ESPript (https://espript.ibcp.fr/ESPript/ESPript/). Protein physicochemical properties were predicted with ProtParam (https://web.expasy.org/protparam/) and HeliQuest (https://heliquest.ipmc.cnrs.fr/cgi-bin/ComputParams.py).

The antimicrobial region of Pecbloodin was predicted using the CAMP_R4_ database (http://www.camp.bicnirrh.res.in/prediction.php). Based on this prediction and several well-established parameters for antimicrobial peptide design—including net positive charge, hydrophobicity, amphiphilicity, α-helical propensity, and an optimal sequence length—a 20-residue fragment, Pecbloodin_18-37_ (H-LYFLIRAVRLKKPAPKKKYG-OH), was chemically synthesized (97.8% purity) by GenScript (Nanjing, China) and stored at −20°C in lyophilized form.

### Tissue distribution and expression analysis by qPCR

Tissues, including blood, spleen, trunk kidney, intestine, brain, head kidney, gills, skin, heart, liver, and muscle, were collected from healthy mudskippers. To assess immune-responsive expression, blood, liver, spleen, and trunk kidney were collected at 0, 6, 12, 24, 48, and 72 h post-infection with 1.0 × 10^4^ CFU/fish of *E. tarda* (13, 40), a sublethal dose that has been widely used to induce measurable innate immune responses without causing excessive mortality or severe tissue damage in mudskipper infection models.

Absolute qPCR was used to determine tissue distribution, while relative qPCR was used to assess temporal expression post-infection. Reactions were performed using a CFX384 real-time PCR system (Bio-Rad, USA) following previously established protocols (44). Relative expression was calculated using the 2^−ΔΔCt^ method (45).

### Antimicrobial activity assay

Antibacterial activity was evaluated using a broth microdilution method (46). Bacterial suspensions (~10^6^ CFU/mL) were incubated with Pecbloodin_18-37_ (3–192 μM) in 96-well plates for 24 h at species-specific optimal temperatures. LL-37 (GL Biochem, Shanghai) was used as a positive control. MIC was defined as the lowest peptide concentration preventing visible growth, and MBC as the concentration causing ≤99.99% reduction in viable cells. All assays were performed in triplicate and repeated three times.

### Time-kill kinetics assay

Time-dependent bactericidal activity of Pecbloodin_18-37_ was assessed against *A. baumannii* and *S. aureus*. Bacteria were prepared in Mueller-Hinton broth (MHB) at ~1 × 10^6^ CFU/mL and incubated with Pecbloodin_18-37_ at 1× and 2× MBC. At predetermined time points, aliquots were collected, serially diluted, and plated on nutrient agar (HKM, China). After incubation at 37°C for 18–24 h, colony counts were recorded. Bactericidal efficacy was expressed as percent CFU = (CFU at time point/initial CFU) × 100%. Each assay was performed in triplicate and repeated three times.

### Thermal stability and sodium ion tolerance

To evaluate thermal stability, Pecbloodin_18-37_ was preheated at 100°C for 0, 20, or 30 min. The heated peptides were then incubated with *A. baumannii* (~1 × 10^6^ CFU/mL) in MHB at 37°C for 24 h. Bacterial growth was quantified by absorbance at 595 nm using a microplate reader.

For sodium ion tolerance, *A. baumannii* cultures were co-incubated with Pecbloodin_18-37_ (1× MIC) in MHB containing NaCl at final concentrations ranging from 20 to 160 mM. After 24 h at 37°C, absorbance at 595 nm was measured. All experiments were performed in triplicate and repeated at least three times.

### Measurement of ROS

Intracellular ROS production was assessed using 2′,7′-dichlorodihydrofluorescein diacetate (DCFH-DA; Jiancheng, China). Bacterial suspensions of *A. baumannii* and *S. aureus* (~1 × 10^8^ CFU/mL) were incubated with Pecbloodin_18-37_ (1.5–12 μM) or LL-37 (12 μM) for 30 min. After washing with phosphate-buffered saline (PBS), cells were treated with DCFH-DA (10 μM) for an additional 30 min. Fluorescence intensity was measured at 488/533 nm using a microplate reader. Each assay included five replicates and was repeated three times.

### SEM analysis

The morphological effects of Pecbloodin_18-37_ were examined using SEM (Zeiss SUPRA 55, Germany) as previously described (47). Bacterial and fungal strains were suspended in 10 mM sodium phosphate buffer (NaPB) to final concentrations of ~1 × 10^8^ and ~1 × 10^7^ CFU/mL, respectively, and treated with Pecbloodin_18-37_ (1× MBC) for 20 min at room temperature. Cells were fixed in 2.5% glutaraldehyde overnight, dehydrated through an ethanol gradient, dried using a critical point dryer (EM CPD300, Leica), gold-coated, and visualized under SEM.

### Membrane permeability assay

The effect of Pecbloodin_18-37_ on bacterial inner membrane integrity was evaluated using the LIVE/DEAD BacLight kit (Thermo Fisher, USA) as previously described (48). Log-phase cultures of *A. baumannii* and *S. aureus* were washed in 10 mM NaPB (pH 7.4) and adjusted to 1 × 10^7^ CFU/mL. Cells were incubated with Pecbloodin_18-37_ (1× MBC) at 37°C for 15 min. PMB (1 μg/mL) served as a positive control. Following staining with SYTO 9 and PI, samples were incubated in the dark for 15 min and analyzed using confocal laser scanning microscopy (Zeiss LSM780, Germany).

### Biofilm inhibition assays

The anti-biofilm activity of Pecbloodin_18-37_ was assessed as previously described (49). Log-phase *A. baumannii* and *S. aureus* cultures (~1 × 10^6^ CFU/mL) were incubated in 96-well plates with varying concentrations of Pecbloodin_18-37_ (0–48 μM) at 37°C for 24 h. Formed biofilms were stained with 0.1% crystal violet (Sigma-Aldrich, Germany), and absorbance at 595 nm was measured. Each experiment included five replicates and was repeated three times.

### Resistance development under long-term exposure

To assess the potential for resistance development, *A. baumannii* and *S. aureus* were serially passaged for 48 days in the presence of sub-MIC levels of Pecbloodin_18-37_, following a modified protocol (50). Conventional antibiotics (gentamicin, tigecycline, vancomycin, and ampicillin) and LL-37 were included for comparison. Each day, cultures were diluted 1:1,000 into fresh MHB containing the respective agent. MIC values were determined periodically. All experiments included three biological replicates and were repeated independently three times.

### Cytotoxicity and hemolytic activity

The cytotoxicity of Pecbloodin_18-37_ toward HEK-293T, HeLa, and ZF4 cells was assessed using the MTS assay (49). Cells (~1 × 10^5^ cells/mL) were seeded into 96-well plates, incubated for 10 h, and then treated with Pecbloodin_18-37_ (6–96 μM) for 24 h. Melittin (6 μM) was used as a positive control. Each assay was performed in quintuplicate and repeated three times.

Hemolytic activity was assessed using freshly isolated mouse erythrocytes. Red blood cells were washed with 0.9% saline and resuspended to a 4% (vol/vol) cell suspension. Aliquots (100 μL) were incubated with 100 μL of Pecbloodin_18-37_ (various concentrations) at 37°C for 1 h. Following centrifugation (3,000 *g* for 4 min), supernatant absorbance was measured at 540 nm. Saline and 0.1% Triton X-100 served as negative and positive controls, respectively. Hemolysis (%) was calculated as:


$$Hemolysis(\%)=\frac{A_{sample}-A_{negative}}{A_{positive}-A_{negative}}\times 100.$$

### *In vivo* protective efficacy in *B. pectinirostris*

The therapeutic efficacy of Pecbloodin_18-37_ was evaluated in *B. pectinirostris* following intraperitoneal challenge with *E. tarda*. Fish (*n* = 40 per group) were injected with 1.3 × 10^7^ CFU/fish of *E. tarda* (a dosage corresponding to the LD_50_). One hour post-infection, treatment groups received Pecbloodin_18-37_ (20 μg/fish, an optimal dose determined by preliminary efficacy and safety trials), while controls received PBS. Mortality was recorded for 48 h, and survival curves were plotted using GraphPad Prism 9.0.

At 48 h, livers were harvested, homogenized, serially diluted, plated on nutrient agar, and incubated at 28°C for CFU quantification. Relative qPCR was conducted to measure the expression of immune-related genes (TNF-α, IL-1β, IL-10, and TLR4) and AMP genes (Hepcidin, LEAP-2, and lysozyme), using *RPL8* as the reference gene. ROS and MPO activity in liver tissue were quantified using commercial kits from Nanjing Jiancheng Bioengineering Institute (China) and Solarbio (China), respectively. These quantitative data were standardized by referencing the uninfected PBS control group. For each parameter, experimental group values were divided by the uninfected control group’s average and presented as fold changes. This uniform approach across all data sets enabled a consistent assessment of infection effects and peptide modulation relative to baseline.

### Statistical analysis

All statistical analyses were conducted using SPSS (version 26.0) and GraphPad Prism (version 9.0). Data are expressed as mean ± standard deviation. Comparisons between two groups were performed using Student’s *t*-test. For multiple group comparisons, one-way ANOVA followed by Dunnett’s or Tukey’s *post hoc* test was applied as appropriate.

In two-group comparisons, statistical significance was indicated as follows: *P < 0.05 and **P < 0.01. For multiple comparisons, different lowercase letters (e.g., *a*, *b*, *c*, and *d*) were used to denote statistically significant differences between groups; groups sharing the same letter were not significantly different (*P* > 0.05).

## Data Availability

All data supporting the findings of this study are publicly available. The *Pecbloodin* cDNA sequence has been deposited in GenBank under accession number OR195701. All remaining data are publicly accessible in the article and its supplemental material.

## SUPPLEMENTAL MATERIAL

Supplemental material aem.02043-25-s0001.docx Table S1; Fig. S1 and S2.

Graphical abstract aem.02043-25-s0002.tiff Visual depiction of the study.

ASM does not own the copyrights to Supplemental Material that may be linked to, or accessed through, an article. The authors have granted ASM a non-exclusive, world-wide license to publish the Supplemental Material files. Please contact the corresponding author directly for reuse.
