# Supplementary material for: Pecbloodin18-37: a promising antimicrobial peptide from Boleophthalmus pectinirostris with therapeutic potential against Edwardsiella tarda infection
Source: Appl Environ Microbiol. 2026 Feb 23;92(3):e02043-25. doi: 10.1128/aem.02043-25 (PMC12997841; doi:10.1128/aem.02043-25)
Supplement: Supplemental material — Table S1; Fig. S1 and S2. [file aem.02043-25-s0001.docx]

**Pecbloodin_18–37_: a promising antimicrobial peptide from *Boleophthalmus pectinirostris* with therapeutic potential against *Edwardsiella tarda* infection**

Yuqi Bai^†1, 2^, Wenbin Zheng^†1, 2^, Weibin Zhang^1, 2^, Jingyuan Zhan^1, 2^, Fangyi Chen^1, 2, 3,^*, Ke-Jian Wang^1, 2, 3,^*

1 State Key Laboratory of Marine Environmental Science, College of Ocean & Earth Sciences, Xiamen University, Xiamen, Fujian, China

2 State-Province Joint Engineering Laboratory of Marine Bioproducts and Technology, College of Ocean & Earth Sciences, Xiamen University, Xiamen, Fujian, China

3 Innovation Research Institute for Marine Biological Antimicrobial Peptide Industry Technology, Fujian Ocean Innovation Center, Xiamen 361102, China

*Corresponding author at: State Key Laboratory of Marine Environmental Science, College of Ocean & Earth Sciences, Xiamen University, Xiamen, Fujian 361102, China.

1. mail addresses: chenfangyi@xmu.edu.cn (F. Chen), wkjian@xmu.edu.cn (K.-J. Wang).

Supplementary Table

**Table S1** Primers used in the study.

| **Primer name** | **Primer sequences (5**′**-3**′**)** | **Comment** |
| --- | --- | --- |
| Pecbloodin-CDS-F | ATGATCCAGCGGGCCCTGTA | Partial fragment amplification |
| Pecbloodin-CDS-R | TCATCTGCGCAGGCTGCGGGC |  |
| Pecbloodin-3′-F1 | GGTGGAGATGGAGGGAGTGGAGAG | 3′RACE |
| Pecbloodin-3′-F2 | GAGGGAGTGGAGAGTGAAGAGGA |  |
| Pecbloodin-5′-R1 | TCACTCTCCACTCCCTCCATCTCCA | 5′RACE |
| Pecbloodin-5′-R2 | GGTTTCTTCAGTCTCACGGCTCGGA |  |
| UPM long | CTAATACGACTCACTATAGGGCAAGCAGTGGTATCAACGCAGAGT | Provided by SMARTer^TM^ RACE cDNA Amplification Kit |
| UPM short | CTAATACGACTCACTATAGGGC |  |
| NUP | AAGCAGTGGTATCAACGCAGAGT |  |
| Pecbloodin-qRT-F1 | GCCGTGAGACTGAAGAAACC | Real-time PCR |
| Pecbloodin-qRT-F2 | ACTCTCCACTCCCTCCATCT |  |

Supplementary Figures


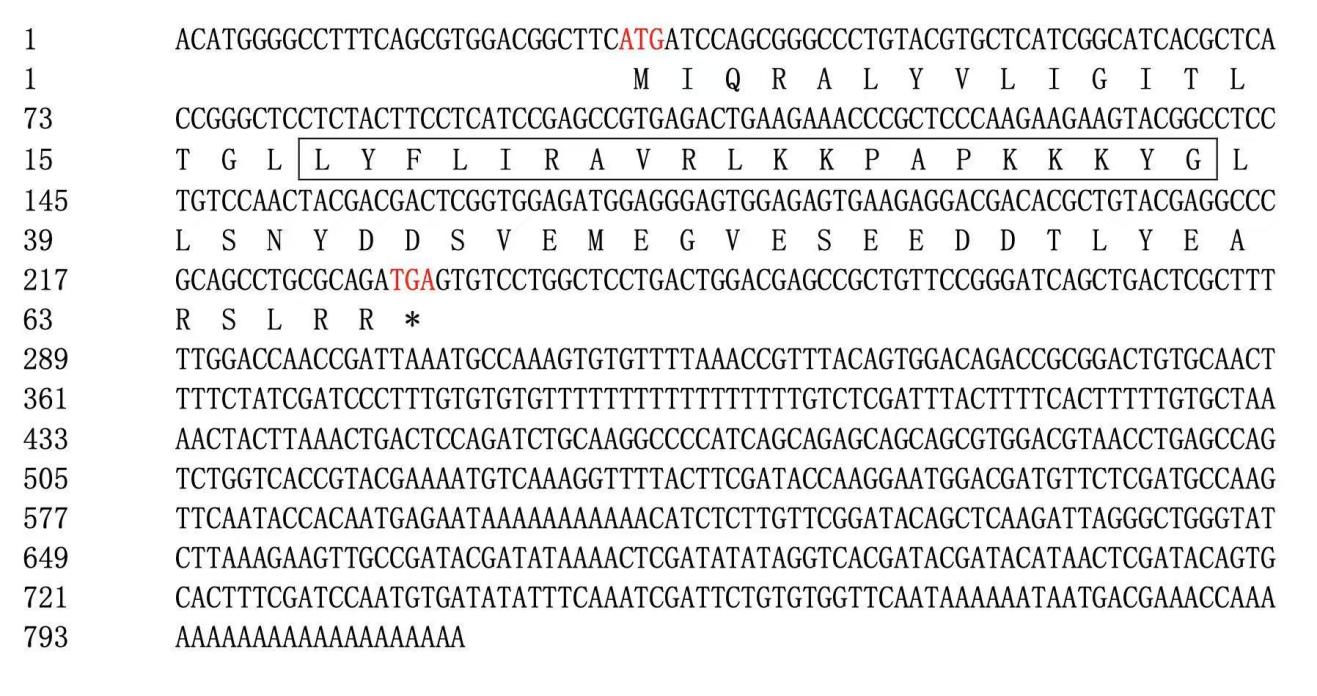


**Fig. S1.** Full-length cDNA and deduced amino acid sequences of *Pecbloodin*. The sequence includes the 5′ and 3′ untranslated regions, coding sequence, and the predicted protein translation.


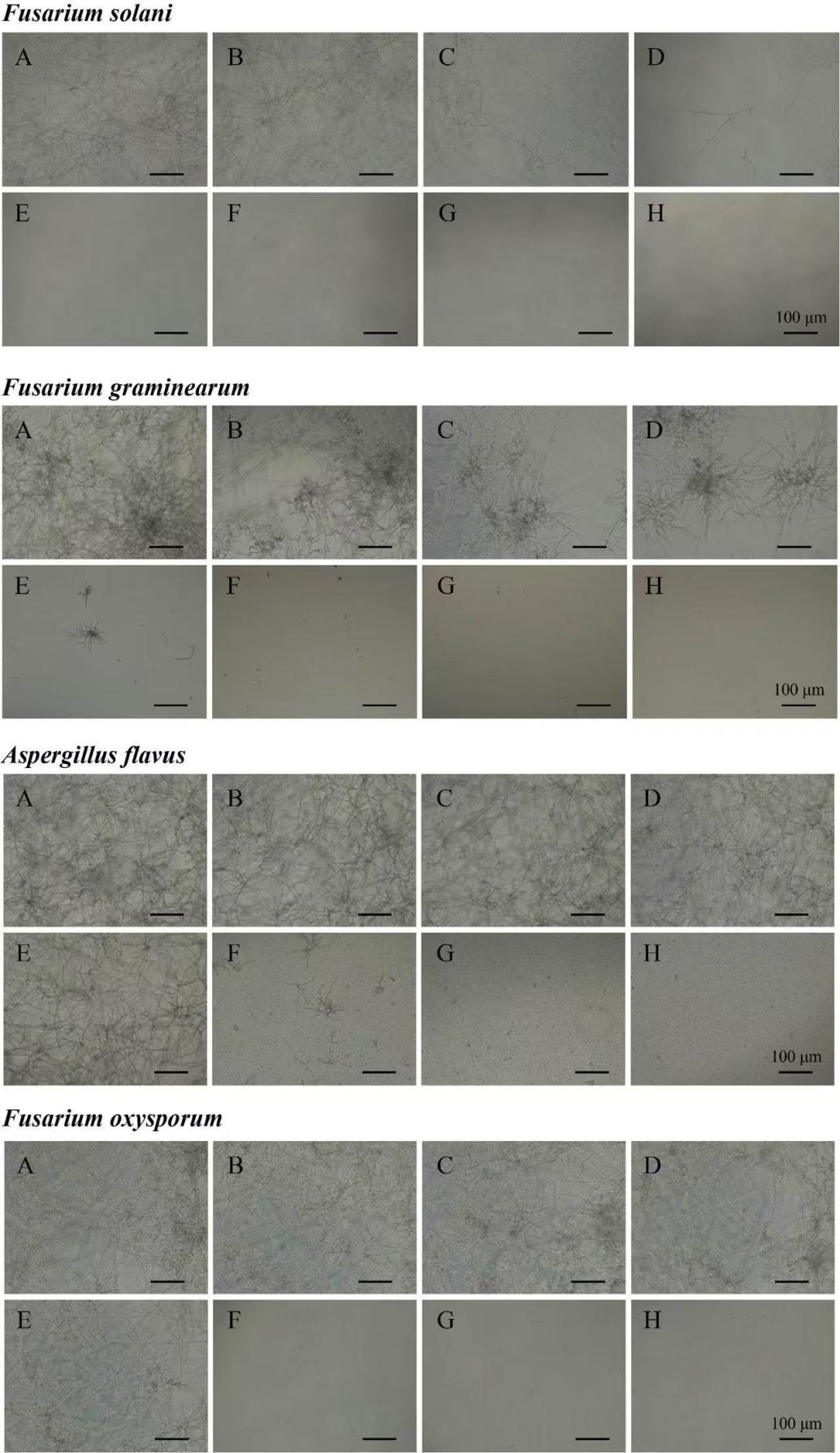


**Fig. S2.** Microscopic observation of the effect of Pecbloodin_18–37_ on mold spore germination. Spore germination was examined under an optical microscope after treatment with varying concentrations of Pecbloodin_18–37_: 0, 1.5, 3, 6, 12, 24, 48, and 96 μM (panels A–H, respectively).
