# Supplementary figures and images for: Pecbloodin18-37: a promising antimicrobial peptide from Boleophthalmus pectinirostris with therapeutic potential against Edwardsiella tarda infection
Source: Appl Environ Microbiol. 2026 Feb 23;92(3):e02043-25. doi: 10.1128/aem.02043-25 (PMC12997841; doi:10.1128/aem.02043-25)

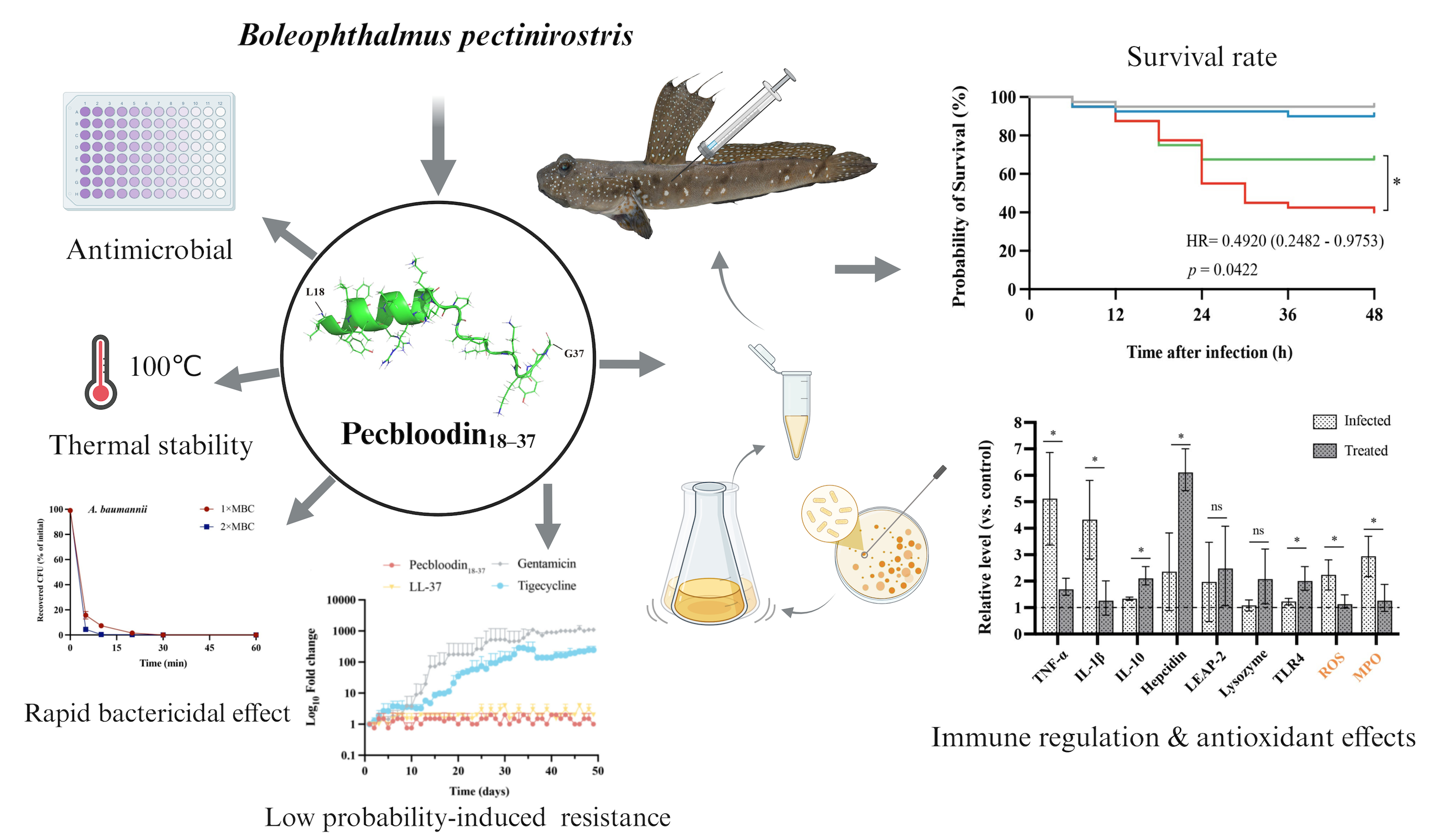

Supplement: Graphical abstract — Visual depiction of the study. [file aem.02043-25-s0002.tiff]
